# Monitoring the Long-Term Molecular Epidemiology of the Pneumococcus and Detection of Potential ‘Vaccine Escape’ Strains

**DOI:** 10.1371/journal.pone.0015950

**Published:** 2011-01-10

**Authors:** Gagan A. Pandya, M. Catherine McEllistrem, Pratap Venepally, Michael H. Holmes, Behnam Jarrahi, Ravi Sanka, Jia Liu, Svetlana A. Karamycheva, Yun Bai, Robert D. Fleischmann, Scott N. Peterson

**Affiliations:** 1 Pathogen Functional Genomics Resource Center, J. Craig Venter Institute, Rockville, Maryland, United States of America; 2 Veterans Affairs Pittsburgh Healthcare System, Pittsburgh, Pennsylvania, United States of America; University of Witwatersrand, South Africa

## Abstract

**Background:**

While the pneumococcal protein conjugate vaccines reduce the incidence in invasive pneumococcal disease (IPD), serotype replacement remains a major concern. Thus, serotype-independent protection with vaccines targeting virulence genes, such as PspA, have been pursued. PspA is comprised of diverse clades that arose through recombination. Therefore, multi-locus sequence typing (MLST)-defined clones could conceivably include strains from multiple PspA clades. As a result, a method is needed which can both monitor the long-term epidemiology of the pneumococcus among a large number of isolates, and analyze vaccine-candidate genes, such as *pspA*, for mutations and recombination events that could result in ‘vaccine escape’ strains.

**Methodology:**

We developed a resequencing array consisting of five conserved and six variable genes to characterize 72 pneumococcal strains. The phylogenetic analysis of the 11 concatenated genes was performed with the MrBayes program, the single nucleotide polymorphism (SNP) analysis with the DNA Sequence Polymorphism program (DnaSP), and the recombination event analysis with the recombination detection package (RDP).

**Results:**

The phylogenetic analysis correlated with MLST, and identified clonal strains with unique PspA clades. The DnaSP analysis correlated with the serotype-specific diversity detected using MLST. Serotypes associated with more than one ST complex had a larger degree of sequence polymorphism than a serotype associated with one ST complex. The RDP analysis confirmed the high frequency of recombination events in the *pspA* gene.

**Conclusions:**

The phylogenetic tree correlated with MLST, and detected multiple PspA clades among clonal strains. The genetic diversity of the strains and the frequency of recombination events in the mosaic gene, *pspA* were accurately assessed using the DnaSP and RDP programs, respectively. These data provide proof-of-concept that resequencing arrays could play an important role within research and clinical laboratories in both monitoring the molecular epidemiology of the pneumococcus and detecting ‘vaccine escape’ strains among vaccine-candidate genes.

## Introduction

*Streptococcus pneumoniae*, a causative agent of pneumonia, meningitis, otitis media and bacteremia causes significant morbidity and mortality worldwide. Young children, the elderly population and immunocompromised individuals are at the highest risk of pneumococcal diseases. *S. pneumoniae* is also the predominant pathogen in community-acquired pneumonia [1] and is associated with substantial health care cost in adults [2]. The 23-valent pneumococcal polysaccharide vaccine (PPV23) provides significant serotype-specific protection against invasive disease in adults; however, rates of invasive pneumococcal disease (IPD) were still substantial. Fortunately, the introduction of the 7-valent pneumococcal protein conjugate vaccine (PCV7) in 2000, which includes serotypes 4, 6B, 9V, 14, 18C, 19F and 23F, led to significant decline in invasive pneumococcal disease in children and adults through receipt of the vaccine and herd immunity, respectively [2], [3].

However, serotype replacement is already eroding the efficacy of PCV7 in high-risk populations [4], [5]. In children, the incidence of non-PCV7 serotype 3 invasive disease is significantly increasing [6], and has been associated with increased morbidity among patients with pneumococcal pneumonia [7]. Moreover, serotype 3 strains have caused an increasing proportion of cases of otitis media in the PCV7 era [8]. The recent licensure of PCV13 [9], which includes PCV7 serotypes plus serotypes 1, 3, 5, 6A, 7F and 19A will likely decrease serotype 3 disease; however, non-PCV13 serotypes may likely be associated with serotype replacement in the future.

Serotype replacement can occur due to the expansion of existing clones, emergence of new clones, or serotype capsular transformation with strains with PCV7 serotypes. Multilocus sequence typing (MLST) is a standard molecular subtyping technique which can ascertain the genetic relatedness between strains [10]. A clone can be defined as sharing 5 of 7 housekeeping gene alleles, or belonging to the same sequence type (ST)-complex. Strains which belong to the same ST-complex but have different serotypes are commonly classified as strains that have undergone capsular transformation. To illustrate, the serotype 3 strains associated with an increasing percentage of otitis media in the PCV7 era all belong to ST180-complex [8], suggesting the expansion of a non-PCV7 clone. Likewise, non-PCV7 serotype 19A disease has dramatically increased in the United States in the PCV7 era, and most of the penicillin-resistant serotype 19A strains appear to have arisen through capsular transformation with the Taiwan ^19F^-14 clone [11]. In both scenarios clonal expansion occurred, reinforcing the theory that a few clones cause a disproportionate amount of pneumococcal disease.

While MLST and serotyping analysis can quickly ascertain strains which have undergone recombination at the capsular locus, additional methods are needed to determine the frequency of SNPs, recombination, and deletions in virulence genes. Understanding the role of recombination and polymorphism in the genetic diversity of an organism, bacterial evolution and emerging pathogens is vital in developing therapeutic or vaccine strategies for a disease [12]. Recombination-mediated genome plasticity is considered to be a strategy for adapting to environmental changes for *S. pneumoniae* and represents a mechanism for rapid evolution of the genome [13] leading to emergence of new genotypes with altered or novel phenotypes [14]. This can have a considerable impact on bacterial evolution and human health. Recombination in house-keeping and virulence genes has been reported in group B Streptococcus (GBS) as a factor contributing towards genomic diversity between genotypes and for distinct disease pathogenesis of strains [15]. Inter and intra-species recombination in *S. oralis* has also been suggested [16].

Pneumococcal genomics has accelerated whole genome comparative analysis of strains and have led to identification of a core set of essential genes, genes contributing to virulence and genes involved in maintaining a non-invasive phenotype [17], [18], [19], [20], [21]. Despite the decreasing cost of complete genome sequencing, this method is cost-prohibitive given that thousands of clinical isolates will need to be sequenced for epidemiologic purposes. Moreover, the software analysis programs cannot determine the significance of each mutation detected As a result, the development of multi-virulence-locus sequence typing (MVLST) scheme has been reported. This method was found to be more suitable for local epidemiology, providing higher level of discrimination for *Listeria monocytogenes* compared to MLST based on housekeeping genes of this species [22]. An alternative to MLST is also needed for *S. pneumoniae*. For example, strains belonging to the same ST or serotype have been shown to differ genetically and phenotypically in an animal model of infection [23]. Moreover, recent studies indicate that a repertoire of accessory regions can differ even among isolates of the same clone [24]. However, MVLST may not be feasible for *S. pneumoniae*, given that up to 10% genome variation exists between *S. pneumoniae* strains [25].

In this study, we developed a targeted Affymetrix, Inc. GeneChip® resequencing approach based on a single TIGR4 reference genome supplemented with Sanger sequencing to determine whether this method correlated with MLST, and whether it could provide further discrimination among highly-related strains. We also sought to determine whether nucleotide-level diversity, recombinations, and deletions could be detected among 72 pneumococcal strains. The five conserved gene sequences include 16S rRNA, DNA-directed RNA polymerase and three hemolysin gene fragments. The 16S rRNA and DNA-directed RNA polymerase are not only conserved sequences but also important housekeeping markers. Both are stable phylogenetic markers widely used in bacterial phylogenetics due to reduced frequency of heterologous gene exchanges [26], [27]. Furthermore, the gene encoding the highly conserved subunit of the bacterial RNA polymerase, has been used to identify streptococcus species of medical interest [28], [29]. Hemolysins are pore-forming exotoxin proteins that cause *in vitro* lysis of red blood cells. In *S. pneumoniae*, these major virulence proteins have been renamed pneumolysins due to their ability to lyse any eukaryotic cell containing cholesterol in its membrane [30]. The hemolysin and hemolysin-related sequences are relatively conserved in *S. pneumoniae* compared to other virulence factors.

The six variable gene sequences encode a portion of four cell wall surface anchor proteins and two pneumococcal surface proteins. Surface proteins play a vital role in the infectious process of pathogenic bacteria and are known to significantly contribute to virulence [31]. Pneumococcal surface protein A (*pspA*), a mosaic gene which has evolved through extensive recombination, is a vaccine candidate [32], [33]. Specifically, PspA interferes with the fixation of complement C3 [34] and binds human lactoferrin [35]. PspA attaches to the pneumococcal cell surface at its C-terminus by binding its conserved choline-binding domain to the choline in the membrane-associated lipoteichoic acid [35]. The proline-rich region is adjacent to the choline-binding domain, and is likely cell wall associated. In contrast, the N-terminal alpha-helical coil, which includes the clade-defining region, is exposed. The clade-defining region, determined by residues 192–290, is markedly diverse. A classification has been adopted that divides PspA molecules into three families and six clades: family 1 includes clades 1 and 2, family 2 includes clades 3,4, and 5, and family 3 includes clade 6 [32]. While PspA clade designation is not associated with serotype, a single clone usually consists of strains from one specific PspA clade [36].

Most of the immunization studies target the clade-defining region within the N-terminal alpha-helical coil on the pneumococcal cell surface, since these epitopes are highly antibody accessible. While vaccines which target a specific clade can provide cross-protection against strains from different clades [37], [38], the highest degree of protection is detected when the challenge strain is from the same clade [39], [40]. Recently, immunization with conserved recombinant proline-rich region of PspA has been demonstrated to provide protection against infection due to strains from different PspA clades [41].

In an effort to ascertain the genetic relatedness of these strains, we developed a resequencing array of five conserved and six variable sequences. In this array, the vaccine candidate evaluated was PspA; however, other arrays could include other variable virulence genes. We anticipated that the accuracy of the array would be ≥90% among conserved sequences, and ≥50% among variable sequences. We also hypothesized that the phylogenetic analysis of the 11 concatenated gene sequences would both correlate with MLST as well as provide further discrimination between strains. Furthermore, we anticipated that the array would provide proof-of-concept that the DNA Sequence Polymorphism program (DnaSP) and the recombination detection program (RDP) could accurately detect SNPs, large mutations, and recombination events among these strains. If successful in this study, we anticipate that resequencing arrays could both monitor the epidemiology of the pneumococcus as well as monitor for ‘vaccine escape’ strains among vaccine-candidate genes.

## Materials and Methods

Genomic DNA of *S. pneumoniae* strains analyzed in this study are shown in **Table S1**. The strains included the reference strain TIGR4, sequenced strains R6, 670 and G54, the first 25 international pneumococcal clones (www.sph.emory.edu/pmen) [42], and 43 strains that caused acute otitis media in children [8]. The dataset included strains from 24 known serotypes/serogroups and 38 different ST-complexes. The strains isolated from children with acute otitis media included genetically related serotype 3 strains, genetically diverse strains belonging to a medley of different serotypes/serogroups and ST-complexes, and genetically related strains which appeared to have undergone capsular transformation (**Tables 1** **and** **2**). Given the limitation of the software used to generate the figures, the clones, the Spanish 23F clone, will be referenced as Spain ^23F^-1 or 23F-1. Genomic DNA was isolated from the strains with Qiagen DNeasy Blood & Tissue Kit (Valencia, CA) and stored at -80°C.

**Table 1:** ST-complex and serotype/serogroup designation of 72 pneumococcal strains.

ST complex	Total number of strains/ST-complex	Serotype/Serogroup (no. strains)
ST81	5	23F (2), 19F (2), 23B (1)
ST180	18	3 (18)
ST199	3	19A (2), 15 (1)
ST377	4	35B (1), 35 (1), 6B (1), 14 (1)
ST690	3	6A (1), 19A (1), NT (1)
ST9	1	14 (1)
ST18	1	14 (1)
ST20	1	14 (1)
ST37	1	23F(1)
ST41	1	19A (1)
ST62	1	11 (1)
ST63	1	15A (1)
ST67	1	14 (1)
ST75	1	19A (1)
ST90	2	6B (2)
ST113	1	18C (1)
ST156	2	9V (2)
ST173	1	23F (1)
ST175	1	19A (1)
ST177	1	19F (1)
ST185	1	6B (1)
ST205	1	4 (1)
ST227	1	1 (1)
ST236	1	19F (1)
ST242	1	23F (1)
ST268	1	19A (1)
ST270	1	6B (1)
ST289	1	5 (1)
ST315	1	6B (1)
ST376	1	6A (1)
ST384	1	6B (1)
ST393	1	38 (1)
ST433	1	22 (1)
ST498	1	35 (1)
ST659	1	16 (1)
ST816	1	10 (1)
ST1201	1	7 (1)
ST1257	1	20 (1)
TIGR4	1	4 (1)
R6	1	2 (1)
G54	1	19F (1)
670	1	6B (1)

**Table 2:** Serotype/Serogroup and ST-complex designation of 72 pneumococcal strains.

Serotype/Serogroup	ST-complexes (no. strains)
1	227 (1)
2	R6 strain
3	180 (18)
4	205 (1) & TIGR4 strain
5	289 (1)
6A	376 (1), 690 (1)
6B	90 (2), 185 (1), 270 (1), 315 (1), 377 (1), 384 (1) & 670 strain
7	1201 (1)
9V	156 (2)
10	816 (1)
11	62 (1)
14	9 (1), 18 (1), 20 (1), 67 (1), 377 (1)
15	199 (1)
15A	63 (1)
16	659 (1)
18C	113 (1)
19A	41 (1), 75 (1), 175 (1), 199 (2), 268 (1), 690 (1)
19F	81 (2), 177 (1), 236 (1) & G54 strain
20	1257 (1)
22	433 (1)
23B	81 (1)
23F	37 (1), 81 (2), 173 (1), 242 (1)
35	377 (1), 498 (1)
35B	377 (1)
38	393 (1)
NT	690 (1)

### Design of *S. pneumoniae* Custom Resequencing Array

Fully sequenced and completely annotated *S. pneumoniae* TIGR4 sequence (GenBank Accession: AE005672) [43] was used as a reference genome to design a 30 Kb CustomSeq resequencing chip. Both the strands of the selected 11 non-contiguous genomic regions were tiled on the array representing ∼0.5 to 5.3 Kb of fragment size for a total of 20,169 bases (**Table 3**). A single *S. pneumoniae* resequencing chip with a standard array format of 12.8 mm and feature size of 20×25 micron design capable of resequencing a maximum of 29,375 bases was fabricated by Affymetrix, Inc. (Santa Clara, CA). A total of 20,169 bp of non-contiguous reference array sequence would provide resequencing coverage of 19,905 bases, corresponding to 0.933% of pneumococcal genome information.

**Table 3:** The eleven genomic fragments included on the TIGR4 resequencing chip

Gene Classification	Name/Locus	Gene/Sequence	Length (bp)	Sequenced length (bp)
Conserved	16S_rRNA	16S rRNA	1413	1389
	SP_0834	Hemolysin-related protein	510	486
	SP_1204	Hemolysin A - putative	594	570
	SP_1466	Hemolysin	645	621
	SP_1961	DNA-directed RNA polymerase – β subunit	3609	3585
Variable	SP_0368	Cell wall surface anchor family protein 1	5301	5277
	SP_1833	Cell wall surface anchor family protein 2	2124	2100
	SP_1992	Cell wall surface anchor family protein 3	663	639
	SP_2145	Antigen, cell wall surface anchor family	2082	2058
	SP_0667	Pneumococcal surface protein - putative	996	972
	SP_0117	Pneumococcal surface protein A	2232	2208
		**Total Bases**	**20169**	**19905**

### Targeted-genome Resequencing Assay

*S. pneumoniae* genomic DNA amplification, DNA fragmentation, labeling, hybridization and acquisition of raw data was carried out as described earlier [44] with minor modifications for 30K resequencing array. Briefly, 2 µg of genomiphied DNA per chip was fragmented in a 50 µl reaction containing 2 µl of fragmentation reagent (0.15U/µl, Affymetrix, Inc., Santa Clara, CA) for 20 minutes at 37°C for the resequencing assay and chips were washed and stained on the GeneChip® fluidics station 450 using the pre-programmed DNA Array_WS2 wash protocol and scanned with GeneChip® Scanner 3000 (Affymetrix, Inc., Santa Clara, CA). The Affymetrix Genechip® DNA Analysis Software (GDAS) Version 3.0.1 with default resequencing algorithm settings for haploid model system was used to analyze hybridization results and obtain raw data. The raw data was processed with our bioinformatic filters. These bioinformatic filters consists of Perl scripts that operate on the CHP files generated by GDAS software and produce a list of high-confidence sequence and SNP calls from a larger raw data set present in those files [44], [45]. These scripts are available for download from our website http://pfgrc.jcvi.org/index.php/compare_genomics/snp_scripts.html.

### Extension of Sequence Coverage

The pneumococcal genome plasticity and inherent limitation of sequencing by hybridization approach resulted in ‘no calls’ on resequencing platform in several pneumococcal strains, especially in rapidly evolving variable gene sequences. In such cases we used ABI Sanger Bigdye terminator sequencing chemistry to expand the coverage of the genomic information obtained from resequencing platform. Sequences obtained from the same regions of multiple strains on resequencing arrays were used to design gene-specific sequencing primers for the genomic fragments. The template DNA from various pneumococcal strains required for ABI sequencing was PCR amplified using end-primers designed using the corresponding TIGR4 reference sequence. Primers (**Table S2)** were designed using Primer3 [46] and the sequencing was done at the Joint Technology Center (http://www.jcvi.org/cms/research/platforms/sequencing/) using high-throughput 1/64 Big Dye terminator reaction protocol. Briefly, the sequencing reactions performed in 384-well plate format contained 20 ng of PCR amplified and purified gene product, 3.2 pmol of primer and 0.125 µl of Big dye terminator reagent in a total volume of 3 µl. The thermal cycling parameters of 96°C for 2 min followed by 50 cycles of 96°C for 10 seconds and 60°C for 4 min were used. The reaction plate was held at 10°C until loaded on 3730xl DNA analyzer.

The individual ABI sequence reads for a given gene from each strain were assembled *de novo* to form contigs using TIGR Assembler [47]. Each of the contigs was aligned by nucmer (http://mummer.sourceforge.net/manual/#nucmer) against the homologous gene sequence from the reference TIGR4 strain to obtain coordinates for the gene sequence of the strain selected in our study. Similar nucmer alignment analysis was also carried out for the sequence generated for the same gene on the resequencing array platform and the reference sequence. The reference-normalized coordinates from both the alignments were used to mutually compare sequences obtained from ABI sequencing and resequencing arrays. When sequences generated from both ABI and resequencing array methods overlapped but differed in a base call, the base with the better quality score (empirically derived ABI and resequencing array Phred equivalent quality scores) was chosen over the other. In cases where a base call was available from only one of the two sequencing platforms, the base was retained in the final sequence provided the Phred equivalent quality score was at least 20. The ABI sequencing resulted in extending the length of the sequence coverage for the genes to ≥95% (95% to 105%) when compared to the corresponding TIGR4 sequences except for the eleven sequences listed in **Table S3** (1.4% of total sequences). All the sequences are available and can be downloaded from our website http://pfgrc.jcvi.org/index.php/compare_genomics/s_pneumo_data_release.html.

The final sequences were aligned by Muscle [48] and alignments were used in the evaluation of the extent of sequence variation, phylogenetic and recombination analyses. The DnaSP software package, version 5.1 (http://www.ub.edu/dnasp/) [35] was used to estimate DNA sequence variation parameters among multi-aligned sequences (**Table 4**). This program can estimate several measures of DNA sequence variation within and between populations, including summary statistics for total number of mutations, Eta (ή), the number of segregating polymorphic sites (S), % GC; diversity parameters such as average pair wise nucleotide difference per site [NucDiversity, π] [49], the average pair wise nucleotide difference per sequence [AvNumDif, k] [50], and thetaG (θG) - the mutation parameter per sequence [51]. The statistics in each group are normalized for the total net number of sites and expressed per 1000 bases as the number of strains and gene sequences available for analysis differed between various groups.

**Table 4:** Analysis of *S. pneumoniae* DNA sequence polymorphism using DnaSP program†.

Group of strains	Number of strains	Number of ST-complexes or serotypes	Total Number of Sites/Net Sites	Total Number of Mutations (Eta)*	Number of Segregating Polymorphic sites (S)*	Nucleotide Diversity (π)*	Average Number of Nucleotide Differences (k)*	Mutation Rate θG*
All - genes^a^	72		21231/9791	71 (696)	66 (649)	0.0010 (0.0097)	9.74 (95.39)	14.67 (143.60)
Conserved - genes^b^	72		6704/5338	27 (146)	27 (145)	0.0005 (0.0028)	2.83 (15.09)	5.64 (30.12)
Variable - genes^c^	72		14465/4451	123 (547)	113 (503)	0.0040 (0.0179)	17.94 (79.86)	25.35 (112.86)
		**Number of** **ST-complexes**						
Serotype 3(and ST-180 complex)^a^	18	1	20322/17816	31 (560)	31 (550)	0.0004 (0.0077)	7.66 (136.53)	9.14 (162.81)
Serotype 6B^a^	8	6	20359/17814	66 (1169)	58 (1037)	0.0013 (0.0231)	23.13 (412.00)	25.31 (450.85)
Serotype 14^a^	5	5	20034/13168	53 (695)	50 (663)	0.0020 (0.0268)	26.75 (352.20)	25.33 (333.60)
Serotype 19A^a^	7	6	20406/13160	75 (983)	65 (852)	0.0021 (0.0279)	27.86 (366.62)	30.49 (401.22)
Serotype 19F^a^	5	3	20186/16655	35 (586)	35 (582)	0.0010 (0.0166)	16.61 (276.60)	16.89 (281.28)
Serotype 23F^a^	5	4	20078/17462	33 (576)	32 (559)	0.0009 (0.0152)	15.18 (265.10)	15.83 (276.48)
		**Number of** **serotypes**						
ST180(and Serotype 3) a	18	1	20322/17816	31 (560)	31 (550)	0.0004 (0.0077)	7.66 (136.53)	9.14 (162.81)
ST 81^a^	5	3	20004/16770	5 (84)	5 (80)	0.0002 (0.0026)	2.61 (43.80)	2.40 (40.32)
ST 199^a^	3	2	19861/18779	24 (443)	24 (442)	0.0008 (0.0157)	15.71 (295.00)	15.73 (295.33)
ST 377^a^	4	4	19841/18797	7 (126)	7 (126)	0.0002 (0.0036)	3.57 (67.17)	3.66 (68.73)
ST 690^a^	3	3	19591/19007	1 (11)	1 (11)	0.0000 (0.0004)	0.39 (7.33)	0.39 (7.33)

a: Sequences were concatenated in the order 16S rRNA, hemolysin-related protein, putative hemolysin A, hemolysin, DNA-directed RNA polymerase – beta subunit, cell wall surface anchor family protein 1, cell wall surface anchor family protein 2, cell wall surface anchor family protein 3, antigen -cell wall surface anchor family protein, pneumococcal surface protein A, putative pneumococcal surface protein.

b: Order of sequence concatenation for 5 conserved genes was 16S rRNA, hemolysin-related protein, putative hemolysin A, hemolysin, DNA-directed RNA polymerase – beta subunit for the analysis.

c: Six variable gene sequences were concatenated in the order cell wall surface anchor family protein 1, cell wall surface anchor family protein 2, cell wall surface anchor family protein 3, antigen -cell wall surface anchor family protein, pneumococcal surface protein A, putative pneumococcal surface protein. for the analysis.

*: Data normalized for net number of sites and expressed per 1000 bases. Raw values are shown in the parentheses.

† : Data generated using DnaSP program, version 5.1.

### Phylogenetic Analysis

Eleven gene sequences of *S. pneumoniae* strains were used as a single concatenated sequence of multiple genes for phylogenetic analysis. The eleven gene sequences from each of the 72 strains were concatenated in the following order: 16S rRNA, hemolysin-related protein, hemolysin A-putative, hemolysin, DNA-directed RNA polymerase – β subunit, cell wall surface anchor family protein 2, cell wall surface anchor family protein 3, Antigen, cell wall surface anchor family protein, *pspA*, cell wall surface anchor family protein 1 and putative pneumococcal surface protein. MrBayes program version 3.1.2 which uses Markov chain Monte Carlo (MCMC) method to approximate the posterior probabilities of trees generated from the aligned sequences [52], [53], was used with the following settings: F81 nucleotide substitution model, equal rates variation across sites, transition/transversion ratio of 2 [Tratiopr  =  beta (2,1)]. The MCMC analysis was run for 100000 generations with chain sampled every 100^th^ generation resulting in 10000 posterior samples. The final parameter values and trees are summarized after discarding 25% (2500) of the posterior sample. The consensus tree is generated and displayed as either a cladogram showing posterior probabilities for each split or a phylogram with associated mean branch lengths.

### Recombination Analysis

Recombination events among the *pspA* gene of the *S. pneumoniae* strains were identified using recombination detection program (RDP) package which incorporates RDP, GENECONV, Maxchi, Chimaera, 3Seq, Bootscan and SiSscan programs [54] to predict recombination signals from aligned DNA sequences. The recombination events were scored as significant only if at least 4 out of 7 individual programs in the package identified the events with a P value of ≤0.01. The recombinant sequences/events from *pspA* gene of pneumococcal strains were further mapped to specific domains of TIGR4 PspA (SP_0117) using the *pspA* gene accession numbers AF071816 and M74122 [32], [55] to determine the domain coordinates for TIGR4 pspA sequence. The recombination events in the *pspA* gene were divided by the following regions: signal peptide sequence upstream of the gene (SP), the first ∼100 residues of the mature protein (A), the transition zone between A and B (A*), the clade-defining region (B), and the proline-rich region (C).

### Analysis of polymorphisms in gene sequences of pneumococcal isolates

MUMmer tool (http://mummer.sourceforge.net/) was used to detect substitutions, insertions and deletions (indels) in the gene sequences of pneumococcal strains. Gene sequence from individual *S. pneumoniae* strains was compared to the corresponding TIGR4 reference gene sequence using MUMmer. The location and the size of polymorphism(s) were identified and grouped based on their size. Strains showing both unique and common indels (shared by more than one strain) were identified.

*In silico* identified deletions in *pspA* gene of strains belonging to ST81-complex, ST180-complex, and ST199-complex were further mapped to specific domains of TIGR4 *pspA* as described above (for recombination events) and were represented on a linear map using genomic visualization software Genvision version 2.0.0.29 (http://www.dnastar.com).

## Results

### Accuracy of the pneumococcal resequencing array

The selected genomic sequences (**Table 3**) were tiled on the chip using *S. pneumoniae* TIGR4 genome sequence as a reference genome as described in the Methods. TIGR4 is a clinical isolate known to be highly invasive and virulent in a mouse model of infection [56] and therefore makes an ideal reference genome for collection of genomic diversity of other clinical pneumococcal isolates. The fully determined genome sequences of pneumococcal strains TIGR4, R6 and 670 were used to determine the resequencing accuracy of the Affymetrix resequencing platform [**Figure S1** (upper panel)]. The overall accuracy of our resequencing platform was 99.8%, corresponding to an average Phred quality score of 27 [57].

**Figure 1:**
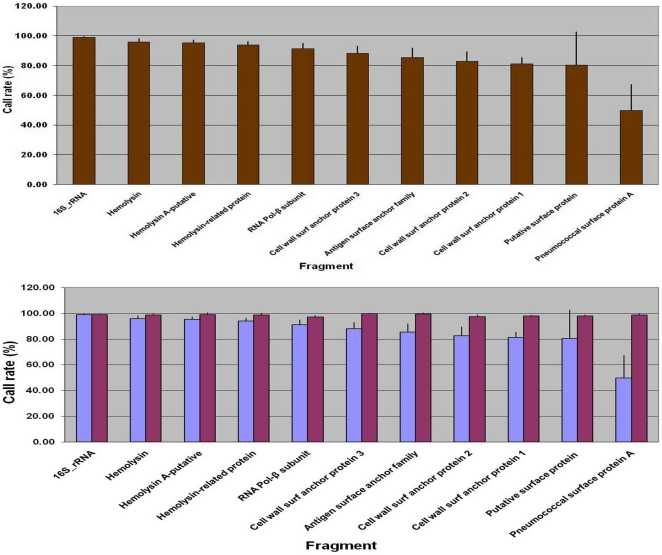
Call rate per pneumococcal genomic fragment. The upper panel shows the resequencing profile of the eleven genomic fragments tiled on the chip and analyzed from 72 strains in duplicates. The variability in call rate increases as the query sequence diverges from the reference on the chip. The vertical bars represent the standard deviation in the results. The lower panel shows the complementation of sequence information using complementary ABI Sanger sequencing method for the genomic fragments. Cumulative data obtained was ≥95% sequence information per fragment (**Table S2**). Light blue bars: resequencing array platform; dark red bars: cumulative data from resequencing array and Sanger sequencing platforms.

We obtained 92 to 99% base call frequency of the conserved genes (16S rRNA, hemolysin, putative hemolysin A, hemolysin-related protein, DNA-directed RNA polymerase - beta subunit) from 72 pneumococci, with the DNA-directed RNA polymerase – β subunit showing the highest variability from among conserved gene fragments. In contrast, the base call frequencies of the variable genes (cell wall surface anchor family proteins 1–3, and antigen, pneumococcal surface protein – putative, *pspA*) ranged from 50 to 90% (**Figure 1** upper panel). **Table S4** summarizes the validation results in terms of the expected SNPs to those observed SNPs using the resequencing platform. The greatest sequence identity between the TIGR4 reference sequence and the query sequence was detected among the conserved genes; while the SNP detection rate was lower in the variable genes, the overall accuracy remained high in these fragments. A higher number of false negatives, especially in variable cell wall surface family proteins and *pspA* genomic regions can be attributed to the sequence diversity together with the stringent filter parameters used [44]. The lower panel in **Figure 1** shows additional sequence coverage (≥95%) for the eleven pneumococcal genomic loci by complementing the resequencing array platform with ABI Sanger sequencing data. As expected, only a small (0.09% to 5.77%) improvement in the sequence information of five conserved housekeeping genes was achieved; in contrast, a significant improvement was obtained in the variable gene sequences, especially in the *pspA* gene where the sequence content increased by almost 50%. TIGR4 shared >99% sequence identity with AF071816, a PspA clade 3 serotype 4 strain (data not shown) [32]. Serotype and ST-complex designations of the 72 strains analyzed are shown in **Tables 1** **&** **2**.

### Phylogenetic analysis

**Figure 2:**
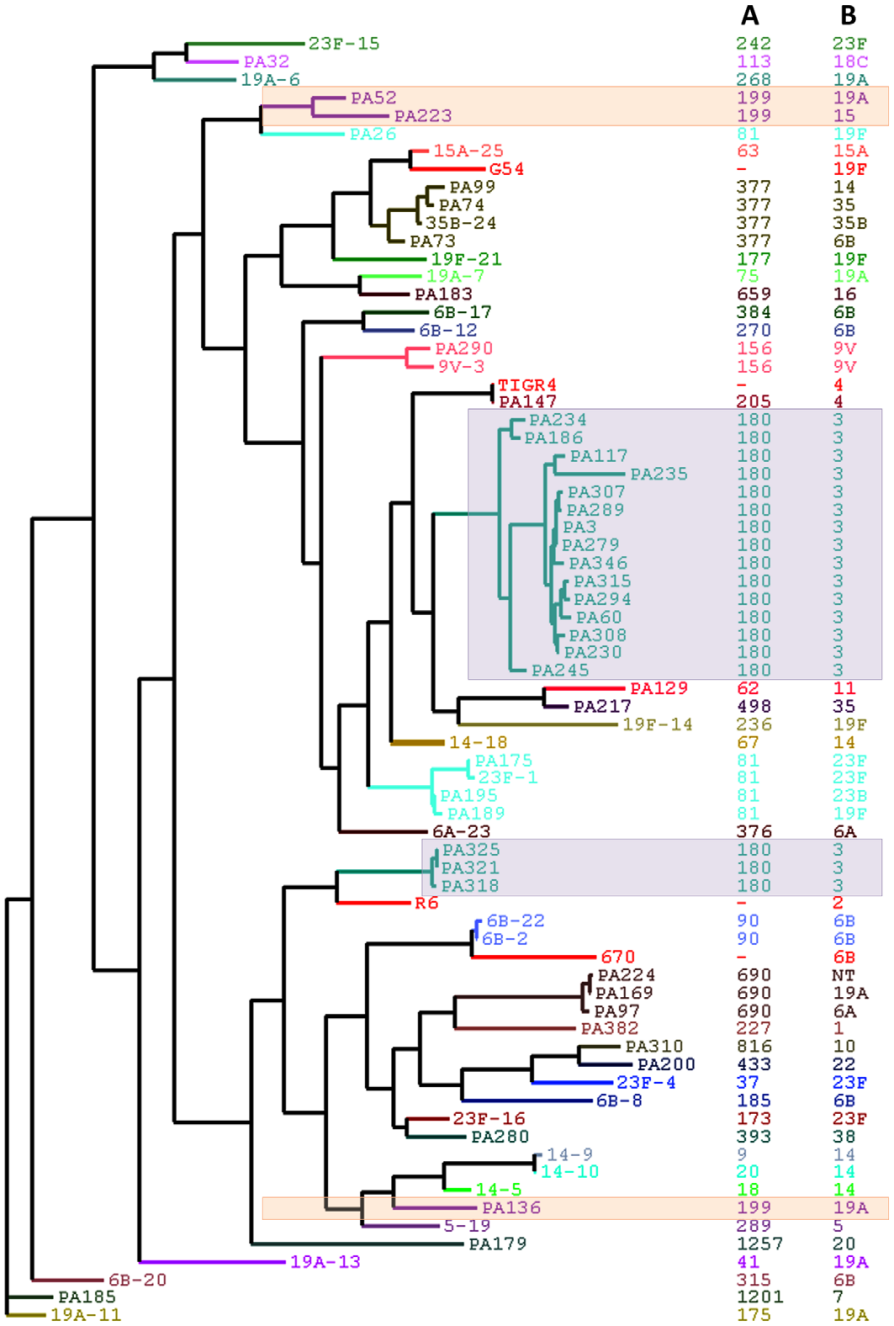
Phylogenetic clustering of pneumococcal strains using concatenated genomic sequences of all 11 genes. Genomic sequence information of all the 11 gene fragments of 72 *S. pneumoniae* strains was used to generate the phylogenetic tree, and MrBayes program was used to generate the consensus tree as described in Methods. The clustering was viewed and edited in TreeDyn http://www.treedyn.org/. The posterior probability score of clusters at its node ranged from 0.5 to 1.0. The frequency of posterior probability score of 1.0 was 63% and only 20% of clusters showed 0.5 as the posterior probability score at their nodes. Unexpected clusters of strains, based on MLST classifications, are indicated with shaded backgrounds. **A**: ST-complex designation, **B**: serotype/serogroup designation.

The phylogenetic clustering of strains based on concatenated genomic sequence information of all the 11 gene fragments is shown in **Figure 2**. In general, the clustering correlated with the ST-complex designation. For example, despite including strains from multiple serotypes, ST690-complex strains clustered together, and ST377-complex strains clustered together. Moreover, all ST690-complex strains were PspA clade 1, with 81.7% sequence identity with respect to a clade-defining region of clade 1 Genbank sequence, AF071804. Likewise, all ST377-complex strains were PspA clade 4, with 88.1–89.1% sequence identity in the clade-defining region to a clade 4 Genbank sequence, AF071824.

Four unexpected clustering patterns were observed. In the first case, two serotype 14 clones clustered together, but did not appear to have related MLST designations. However, further analysis revealed that the England ^14−^9 clone (ST9) and the Slovakia ^14−^10 clone (ST20) have 4 of 7 MLST alleles in common. Moreover, both serotype 14 international clones belong to PspA clade 1, and have identical *pspA* gene sequences. Therefore, their close proximity in phylogenetic clustering is likely an accurate representation of their genetic relatedness. In the remaining three cases, a single MLST-defined clone was divided into two distinct clusters in our tree. Among ST180-complex serotype 3 strains, 75% (3/4) of the strains from the latter part of the 2002 (PA318, PA321, PA325) were grouped in a separate cluster. Among the ST81-complex strains, one strain (PA26) did not cluster with the remaining four strains (^23F^-1, PA175, PA189, PA195). Likewise, among ST199-complex strains, one strain (PA136) did not cluster with the other two strains (PA52, PA223). Strains belonging to both ST81- and ST199-complexes were associated with multiple serotypes, but the clustering in our tree did not correlate with specific serotypes serotype.

**Figure 3:**
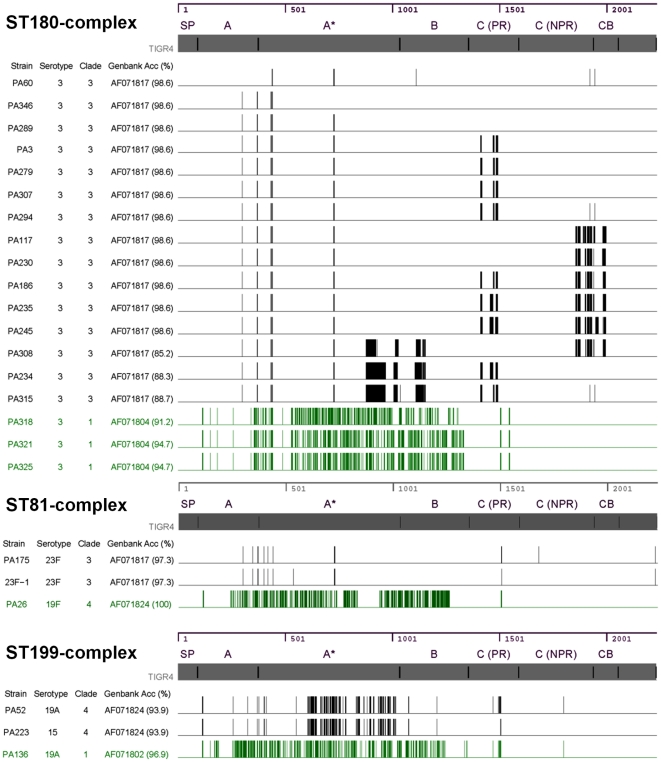
*In silico* mapping of *pspA* gene deletions to protein domains. Deletions in *pspA* gene identified *in silico* for *S. pneumoniae* strains belonging to ST180, ST81 and ST199-complex were used for this analysis. Data for eighteen strains belonging to ST180 (**upper panel**), three strains of ST81-complex (**middle panel**), and three strains of ST199-complex are shown (**lower panel**). Two strains of ST81-complex (PA195 and PA189) were excluded because of low sequence coverage (**Table S3**). The nucleotide coordinates and protein domains of the *pspA* gene are shown at the top of each panel. The thickness of the vertical line represents the size of the deletion. SP: signal peptide, A: ∼first 100 residues of mature protein, A*: transition zone between A and B, B: clade-defining region, C: proline rich (PR) and non-proline rich (NPR) regions, CB: choline binding domain.

Further analyses revealed that the subclusters within an ST-complex were due in part to a variation in the *pspA* gene. As seen in **Figure 3**, some of the strains within an ST-complex had a significantly different pattern of deletions throughout their *pspA* gene sequence when compared to the remainder of the strains within the specific ST-complex. Using the Genbank sequences to analyze the clade-defining region [32], the marked variation that was detected in the *pspA* gene sequence correlated with a different clade designation. For example, unlike most of the ST180-complex strains, which were classified as clade 3 (based on sequence analysis of the clade-defining region), three of the strains collected in 2002 belonged to clade 1. Likewise, the ST81-complex strains belonged to both clades 3 and 4, while ST199-complex strains belonged to clades 1 and 4. Moreover, strains belonging to ST81-complex and ST199-complex appeared to have undergone recombination at both the capsular locus and within the *pspA* gene. For example, PA26 is a serotype 19F variant of the Spain ^23F^-1 clone; however, this clone belongs to PspA clade 3, and PA26 belongs to a different PspA clade and family. Likewise, PA136 and PA223 strains both belong to ST199-complex, but differ by serotype and PspA clade/family classification (**Figure 3**).

### SNP analysis

In an effort to ascertain whether the resequencing data could accurately classify various groups of genes and strains, we compared the total number of SNPs, degree of heterogeneity using DnaSP, and frequency of recombination with the RDP package. **Table 5** shows the quantitative SNP profile of the targeted genomic regions among pneumococcal isolates. The gene sequences classified as ‘conserved’ had fewer SNPs compared to the ‘variable’ gene sequences with one exception. The cell wall surface anchor family protein 2 gene sequence had fewer SNPs than the DNA-directed RNA polymerase, beta subunit. The *pspA* gene was clearly the most polymorphic gene fragment among the variable gene sequences; however, other less well-characterized surface proteins were also quite genetically diverse. The cumulative *S. pneumoniae* SNP profile for all the eleven gene fragments is shown in **Figure S2**. **Table 4** shows the results of the SNP analysis based on the DnaSP program. The %GC content of the sequences ranged from 42–48%. The nucleotide diversity (π) and the mutation rate per sequence (θG) were 8-fold and 4.5-fold higher respectively for the variable gene sequences compared to the conserved sequences.

**Table 5:** Quantitative SNP profile of targeted genomic regions among pneumococcal strains.

Name/Locus	Gene Classification	Gene/Sequence	Length (bp)	No. of strains†	Total SNPs	SNPs/Kb/strain	Fold difference*
16S_rRNA	Conserved	16S rRNA	1413	72	124	1.22	1.00
SP_0834		Hemolysin-related protein	510	72	393	10.70	8.78
SP_1204		Hemolysin A - putative	594	72	165	3.86	3.17
SP_1466		Hemoylsin	645	72	115	2.48	2.03
SP_1961		DNA-directed RNA polymerase, beta subunit	3609	72	1344	5.17	4.24
SP_0368	Variable	Cell wall surface anchor family protein 1	5301	69	6862	18.76	15.39
SP_1833		Cell wall surface anchor family protein 2	2124	71	598	3.97	3.26
SP_1992		Cell wall surface anchor family protein 3	663	72	331	6.93	5.69
SP_2145		Antigen, cell wall surface anchor family	2082	72	2733	18.23	14.96
SP_0667		Pneumococcal surface protein - putative	996	69	798	11.61	9.53
SP_0117		*pspA* ±	2232	68	15240	100.41	82.38

† strains with ≥95% sequence coverage.

*compared to 16S rRNA.

± pneumococcal surface protein A.

We used serotype 3 strains as a point of reference to perform serotype-specific and ST-complex-specific analyses. All serotype 3 strains belonged to one ST180-complex; in contrast strains from other common serotypes each included strains belonging to 3–6 different ST-complexes. Likewise, the ST180-complex was comprised of only serotype 3 strains; in contrast, other ST-complexes were comprised of strains from 2–4 different serotypes. We compared serotype3/ST180-complex strains to other serotypes and ST-complexes that included ≥3 strains, namely ST81, ST180, ST199, ST377, and ST690 (**Table 1**).

Compared to the serotype 3 strains, the nucleotide diversity (π) and mutation rate per sequence (θG) were ≥2-fold higher for serotypes 6B, 14, 19A, 19F, and 23F strains. For example, serotype 3 strains had a nucleotide diversity (π) of 0.0004, and mutation rate per sequence (θG) of 9.14. Strains from serotypes 6B, 14, 19A, 19F, and 23F had nucleotide diversity values (π) ranging between 0.009–0.021, and mutation rates per sequence (θG) ranging between 15.83–30.49. In essence, serotypes associated with more than one ST complex had a larger degree of sequence polymorphism than a serotype associated with one ST complex (**Table 4**).

In general, less sequence polymorphism was detected among strains of the same ST-complex compared to strains of the same serotype (**Table 4**). This association was often true even when strains belonging to the same ST-complex were of different serotypes. For example, ST180-complex strains, consisting of only serotype 3 strains, had 20-fold higher mutation rate than ST690-complex strains, which included strains from serotypes 6A, 19A, and nontypeable (NT) serotypes. One key exception was ST199-complex, consisting of serogroup 15 and serotype 19A strains. The ST199-complex strains were significantly more genetically diverse than the strains belonging to other ST-complexes.

### Recombination analysis of the *pspA* gene

**Figure 4:**
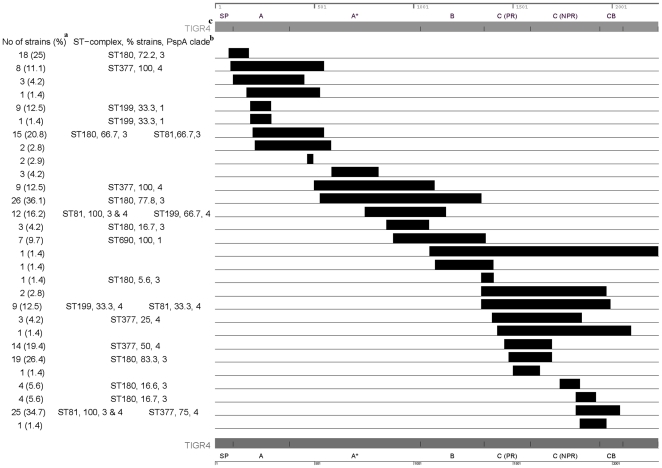
Recombination events detected in the *pspA* gene of 72 strains using the recombination detection package. Two recombination events are not shown in the figure as they could not be mapped to the corresponding TIGR4 PspA sequence. One of these events was detected only in one strain PA179 with nucleotide coordinates 178 to 389. The second event with coordinates 1128 to 1178 was detected in five strains. Two strains of ST81-complex (PA195 and PA189) were excluded because of low sequence coverage (**Table S3**). The nucleotide coordinates and protein domains of the *pspA* gene are shown at the top and bottom of the figure. ^a^ Number (percent) of 72 strains with this recombination event. ^b^ Among ST-complexes that included ≥3 strains: percent of strains belonging to specific ST-complex with recombination event, PspA clade designation(s). ^c^ SP: signal peptide, A: ∼first 100 residues of mature protein, A*: transition zone between A and B, B: clade-defining region, C: proline rich (PR) and non-proline rich (NPR) regions, CB: choline binding domain.

As shown in **Figure 4**, 31 recombination events in the *pspA* gene were detected in the 72 strains using the RDP package. The recombination events spanned from 1–3 domains of the *pspA* gene with 32.3% (10/31) of the events including the clade-defining region, 35.5% (11/31) of the events including the proline-rich region, and 48.4% (15/31) spanning both domains. Strains belonging to ST-complexes containing ≥3 strains were commonly associated with recombination events in the *pspA* gene. In general, recombination events that included the clade-defining region correlated with the PspA designation of those strains. For example, given that strains from ST81-complex, ST180-complex, and ST199-complex belonged to multiple PspA clades, not all of the strains belonging to these ST-complexes would be expected to be implicated in a clade-specific recombination event. The one exception was a recombination event in the clade-defining region that included both PspA clade 3 and clade 4 strains belonging ST81-complex. One other recombination event included both PspA clade 3 and clade 4 strains belonging to the ST81-complex; however, this recombination event spanned the non-proline rich region and the choline binding domain rather than the clade-defining region.

## Discussion

The licensure of the PCV7 vaccine resulted in a marked decline in invasive pneumococcal disease [58], and further reductions are anticipated with the introduction of PCV13. However, since both of these vaccines are serotype-dependent, serotype replacement remains a major concern. For example, non-PCV7 variants of the PCV7 international clones, such as the 19A variant of the Taiwan ^19F^-14 clone, have expanded since introduction of the PCV7 vaccine. Thus the international clones, which by definition are widely disseminated, can evade a conjugate vaccine by capsular transformation. While MLST is a powerful method to establish the genetic relatedness of strains over long periods of time, this method cannot discriminate between highly-related strains of different serotypes or different PspA clades. Variable loci have been suggested to provide higher discrimination than MLST and therefore be more appropriate for local epidemiology and clinical settings [10], [59]. Studies have also been reported where antigen or surface-associated gene sequences have been used with MLST data to infer local epidemiology [36], [60], [61] In this study, we assessed the accuracy of a targeted Affymetrix, Inc. GeneChip® resequencing array for five conserved and six variable genes that encoded conserved proteins, such as hemolysins, and variable surface proteins, including the *pspA* gene. After supplementing the array with Sanger sequencing of diverse regions, the 11 concatenated gene sequences were used in a phylogenetic analysis and compared to the MLST-defined clones to determine if this approach could further discriminate between genetically related strains. Our resequencing array design based only on a single pneumococcal genome (TIGR4) required supplementation of sequencing efforts, especially in the variable gene sequences. Future resequencing array design based on genomic sequence information from a large number of variable gene sequences available in Genbank is likely to eliminate or significantly reduce the need for the complementary Sanger sequencing approach. Moreover, we demonstrated that DnaSP and RDP could accurately identify SNPs and recombination events respectively suggesting, this method could be customized for any set of gene sequences to quickly characterize novel genes and monitor the pneumococcus for ‘vaccine escape’ strains.

The resequencing accuracy of the Affymetrix platform ranged from 92–99% for the conserved gene sequences. As expected, the array could not accurately sequence diverse regions of the variable genes, such as the *pspA* gene; however, the conserved portion of diverse genes could be used to design primers, thereby decreasing the sequencing effort to some degree. In general, the phylogenetic analysis of the 11 concatenated gene sequences created clusters that correlated with the ST-complex designations. On three occasions, an MLST-defined clone was divided into two separate clusters, or subclusters, using the dual platform method. Further analyses revealed that each of the subclusters consisted of a group of strains belonging to the same ST-complex and identical PspA clade designation.

It would be ideal if all strains belonging to a single MLST-defined clone could be targeted by a PspA vaccine. However, these data suggest that commonly-occurring clones include strains from multiple PspA clades and families. Therefore, a PspA vaccine which preferentially protects against one PspA family may be unable to prevent infections due to all strains belonging to a single clone. Specifically, strains belonging to two different PspA clades, and even families, were detected within strains belonging to the Spain ^23F^-1 clone, and clones associated with serotype 3, 15B/C, and 19A strains in the post-PCV7 era [62]. While limited by the small numbers of strains, it is possible that a ST180-complex clone was expanding at the latter part of the study with a different PspA clade, as most of the 2002 strains belonging to this ST-complex were of PspA clade 1 rather than PspA clade 3. In some cases, recombination events involving both the capsular locus and the *pspA* gene were detected in a single strain belonging to a common clone, underscoring the pneumococcus' ability to potentially evade both PCV13 and a PspA vaccine. For example, a child immunized with PCV7, PCV13, and a PspA vaccine containing family 1 (clades 1–3) antigens, could potentially still develop IPD upon exposure to a strain belonging to ST199-complex as this strain is a serogroup 15 strain with PspA clade 4.

In this study, we also sought to determine whether the combined approach of resequencing array complemented with standard sequencing could be used to characterize novel genes and monitor for ‘vaccine escape’ strains through mutations and recombination events. The sequence polymorphism analysis program, DnaSP, confirmed that substantially fewer SNPs were detected in most of the conserved genes compared to the variable genes, with the θG value an excellent marker of genetic diversity. Moreover, the DnaSP program usually detected greater sequence diversity among strains of a given serotype than strains of a given ST-complex. Finally, most of the serotypes which included strains that belonged to multiple ST-complexes had higher sequence heterogeneity than serotypes which included strains belonging to a single ST-complex. As reported previously [32], the RDP data demonstrated that the *pspA* gene had undergone extensive recombination, and the size of the recombination event could span one or multiple protein domains. Individual recombination events varied in frequency of occurrence, ranging from 1–35%. Moreover, strains from ST-complexes including at least 3 strains were commonly implicated. In general, recombination in the clade-defining region correlated with PspA designation, suggesting that these recombination events were accurately identified. Approximately 50% of the recombination events spanned either the clade-defining region or the proline-rich block. Since these two regions are targeted in most of the *pspA* vaccine studies, these data suggest that recombination events in this region could potentially allow the pneumococcus to evade a *pspA*-based vaccine. Taken together, the DnaSP and RDP programs are robust and can be used to characterize novel genes and vaccine-candidate genes from a large number of strains that were analyzed.

In summary, this pneumococcal resequencing array accurately and efficiently resequenced conserved sequences. The resequencing of the variable sequences on the array was comparatively less efficient than the conserved ones; however, the array enabled efficient primer design for the diverse regions, which were subsequently sequenced by an alternate method. Moreover, the phylogenetic analysis both correlated with MLST and detected strains within a single clone that belonged to multiple PspA clades and families. In addition, the array accurately identified both SNPs and recombination events using the DnaSP and RDP programs, respectively.

Future resequencing array designs could include gene sequences from a large number of available *S. pneumoniae* genomes rather than just one genome (TIGR4). By exploiting the large number of available *S. pneumoniae* genomes one could design a comprehensive molecular subtyping tool that does not require a complementary sequencing approach. While there are a plethora of potential pneumococcal targets for future arrays, the inclusion of MLST gene sequences in addition to diverse gene sequences would allow the strain to be simultaneously subtyped by MLST while characterizing clinically-important and biologically-relevant genes. In the future, the resequencing array may become an indispensable molecular subtyping method which can accurately determine the role of recombination and polymorphisms in the evolution of bacteria, such as *S. pneumoniae*, in response to vaccine pressure or antibiotic use.

## Supporting Information

Figure S1 **Resequencing accuracy of** ***S. pneumoniae*** **genomic fragments.** The resequencing accuracy was determined based on the fully sequenced R6 and 670 genomes. The upper panel represents the accuracy of the calls and the lower panel shows the filtered call rates of the fragments for R6 and 670 strains. Call rate is defined as the fraction of total queried bases that can be clearly detected and identified or “called” by the algorithm. The accuracy was higher for 670 sequences compared to R6 genomic fragments. The resequencing accuracy for R6 was found to be 100% for 8 fragments which included all the conserved genes, cell wall surface anchor family proteins 2 and 3 as well as pneumococcal putative surface protein among variable sequences. The resequencing accuracy for two other variable genes cell wall surface anchor family protein 1 and antigen, cell wall surface anchor protein was ≥99.9%. Pneumococcal surface protein A having an accuracy of 97.1%. The resequencing accuracy for the 670 genomic fragments ranged from 98.6 to 100% with nine fragments having 100% accuracy. The resequencing accuracy for two variable genes cell wall surface anchor family protein 1 and pneumococcal surface protein A was ≥99.9% and 98.6% respectively. There are differences in the call rates of the two strains indicating sequence diversity as shown in the lower panel. The pneumococcal putative surface protein (SP_0667) had significantly lower call rate in 670 compared to R6.
(TIF)

Figure S2 **Single nucleotide polymorphisms per** ***S. pneumoniae*** **genomic fragment.** The number of SNPs per genomic fragment were obtained from the sequence data generated using resequencing array (light blue bars) in duplicates and complemented with Sanger sequencing (dark red bars) from all 72 strains (**Table S1**). The validation of resequencing array based SNP detection was done using fully determined genome sequences of TIGR4, R6 and 670 strains **(Table S4).**
(TIF)

Table S1 **Gene sequences of pneumococcal strains (re)sequenced in this study.**
(DOC)

Table S2 **List of primers used for expansion of sequence coverage.**
(DOC)

Table S3 **Gene loci and strains with <95% sequence coverage.**
(DOC)

Table S4 **Validation of resequencing array based on ability to detect SNPs.**
(DOC)
