# Supplementary figures and images for: Monitoring the Long-Term Molecular Epidemiology of the Pneumococcus and Detection of Potential ‘Vaccine Escape’ Strains
Source: PLoS One. 2011 Jan 10;6(1):e15950. doi: 10.1371/journal.pone.0015950 (PMC3018475; doi:10.1371/journal.pone.0015950)

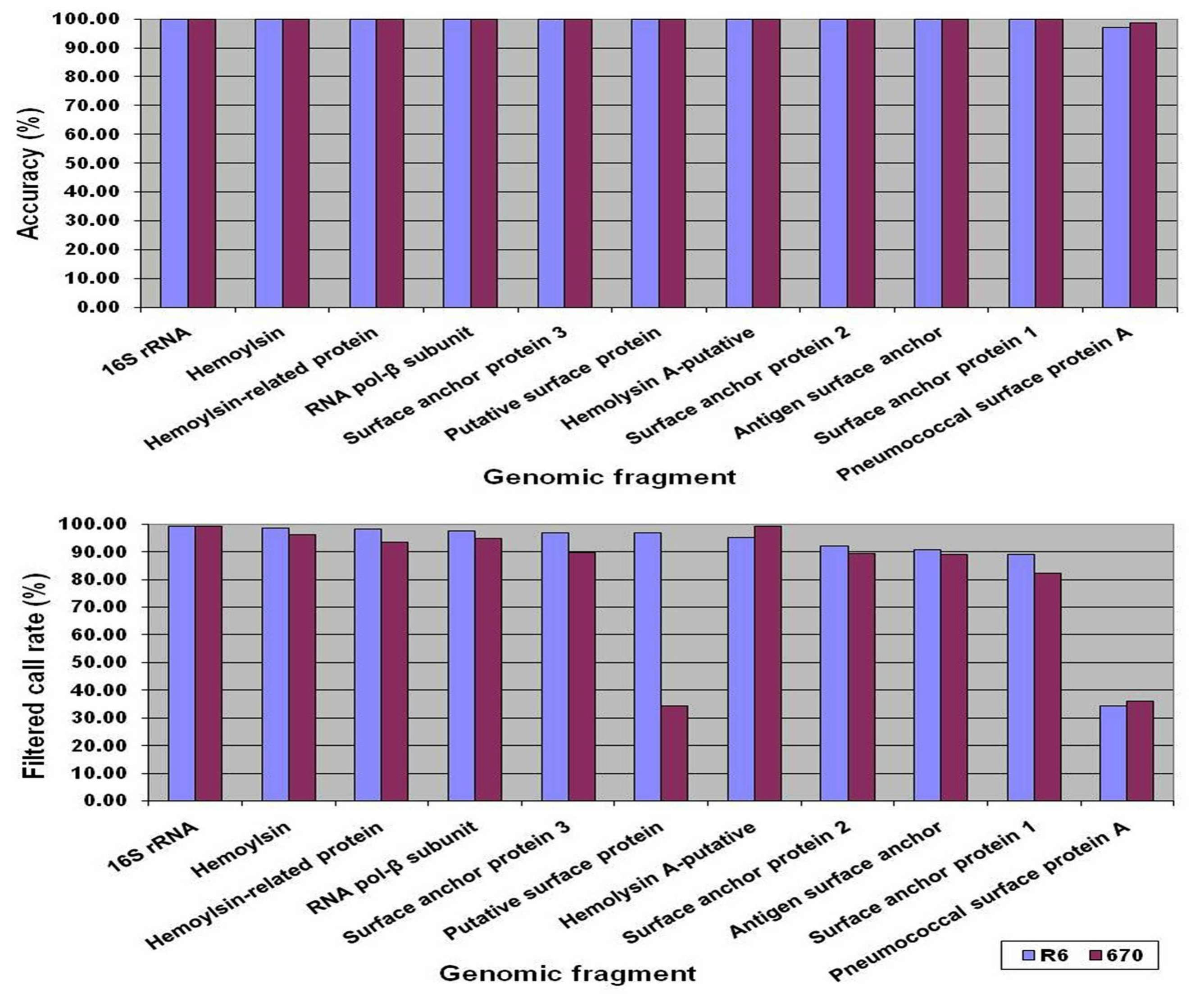

Supplement: Figure S1 — Resequencing accuracy of S. pneumoniae genomic fragments. The resequencing accuracy was determined based on the fully sequenced R6 and 670 genomes. The upper panel represents the accuracy of the calls and the lower panel shows the filtered call rates of the fragments for R6 and 670 strains. Call rate is defined as the fraction of total queried bases that can be clearly detected and identified or “called” by the algorithm. The accuracy was higher for 670 sequences compared to R6 genomic fragments. The resequencing accuracy for R6 was found to be 100% for 8 fragments which included all the conserved genes, cell wall surface anchor family proteins 2 and 3 as well as pneumococcal putative surface protein among variable sequences. The resequencing accuracy for two other variable genes cell wall surface anchor family protein 1 and antigen, cell wall surface anchor protein was ≥99.9%. Pneumococcal surface protein A having an accuracy of 97.1%. The resequencing accuracy for the 670 genomic fragments ranged from 98.6 to 100% with nine fragments having 100% accuracy. The resequencing accuracy for two variable genes cell wall surface anchor family protein 1 and pneumococcal surface protein A was ≥99.9% and 98.6% respectively. There are differences in the call rates of the two strains indicating sequence diversity as shown in the lower panel. The pneumococcal putative surface protein (SP_0667) had significantly lower call rate in 670 compared to R6. (TIF) [file pone.0015950.s001.tif]

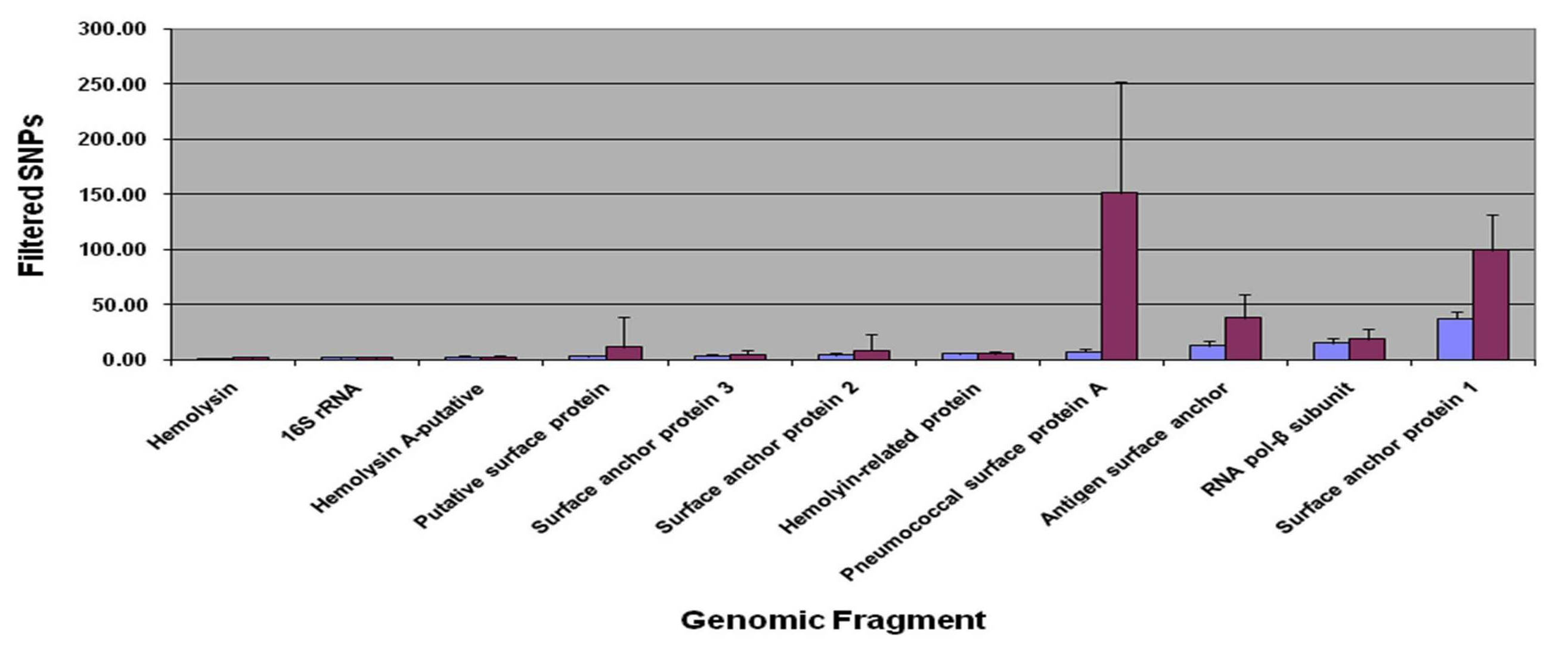

Supplement: Figure S2 — Single nucleotide polymorphisms per S. pneumoniae genomic fragment. The number of SNPs per genomic fragment were obtained from the sequence data generated using resequencing array (light blue bars) in duplicates and complemented with Sanger sequencing (dark red bars) from all 72 strains (Table S1). The validation of resequencing array based SNP detection was done using fully determined genome sequences of TIGR4, R6 and 670 strains (Table S4). (TIF) [file pone.0015950.s002.tif]
