# Supplementary material for: Monitoring the Long-Term Molecular Epidemiology of the Pneumococcus and Detection of Potential ‘Vaccine Escape’ Strains
Source: PLoS One. 2011 Jan 10;6(1):e15950. doi: 10.1371/journal.pone.0015950 (PMC3018475; doi:10.1371/journal.pone.0015950)
Supplement: Table S1 — Gene sequences of pneumococcal strains (re)sequenced in this study. (DOC) [file pone.0015950.s003.doc]

**Table S**1: Gene sequences of pneumococcal strains (re)sequenced in this study

| **S. No** | **Strain** | **Serotype/Serogroup** | **ST-complex** | **Penicillin susceptibility** | **Reference or sample origin** |
| --- | --- | --- | --- | --- | --- |
| 1 | TIGR4 | 4 |  |  | [**43]** |
| 2 | R6 | 2 |  |  |  |
| 3 | G54 | 19F |  |  |  |
| 4 | 670 | 6B |  |  |  |
| 5 | ^14^-9 | 14 | 9 | S | England ^14^-9 |
| 6 | ^14^-5 | 14 | 18 | NS | Spain ^14^-5 |
| 7 | ^14^-10 | 14 | 20 | NS | Slovakia ^14^-10 |
| 8 | ^23F^-4 | 23F | 37 | NS | Tennessee ^23F^-4 |
| 9 | ^19A^-13 | 19A | 41 | NS | S. Africa ^19A^-13 |
| 10 | PA129 | 11 | 62 | S | [8] |
| 11 | ^15A^-25 | 15A | 63 | S | Sweden ^15A^-25 |
| 12 | ^14^-18 | 14 | 67 | NS | Tennessee ^14^-18 |
| 13 | ^19A^-7 | 19A | 75 |  | S. Africa ^19A^-7 |
| 14 | ^23F^-1 | 23F | 81 | NS | Spain ^23F^-1 |
| 15 | PA26 | 19F | 81 | NS | [8] |
| 16 | PA175 | 23F | 81 | NS | [8] |
| 17 | PA189 | 19F | 81 | NS | [8] |
| 18 | PA195 | 23B | 81 | NS | [8] |
| 19 | ^6B^-2 | 6B | 90 | NS | Spain ^6B^-2 |
| 20 | ^6B^-22 | 6B | 90 | S | Greece ^6B^-22 |
| 21 | PA32 | 18C | 113 | S | [8] |
| 22 | ^9V^-3 | 9V | 156 | NS | Spain ^9V^-3 |
| 23 | PA290 | 9V | 156 | S | [8] |
| 24 | ^23F^-16 | 23F | 173 | NS | Poland ^23F^-16 |
| 25 | ^19A^-11 | 19A | 175 | NS | Slovakia ^19A^-11 |
| 26 | ^19F^-21 | 19F | 177 | S | Portugal ^19F^-21 |
| 27 | PA230 | 3 | 180 | S | [8] |
| 28 | PA117 | 3 | 180 | S | [8] |
| 29 | PA186 | 3 | 180 | S | [8] |
| 30 | PA234 | 3 | 180 | S | [8] |
| 31 | PA235 | 3 | 180 | S | [8] |
| 32 | PA245 | 3 | 180 | S | [8] |
| 33 | PA279 | 3 | 180 | S | [8] |
| 34 | PA289 | 3 | 180 | S | [8] |
| 35 | PA294 | 3 | 180 | S | [8] |
| 36 | PA3 | 3 | 180 | S | [8] |
| 37 | PA307 | 3 | 180 | S | [8] |
| 38 | PA308 | 3 | 180 | S | [8] |
| 39 | PA315 | 3 | 180 | S | [8] |
| 40 | PA318 | 3 | 180 | S | [8] |
| 41 | PA321 | 3 | 180 | S | [8] |
| 42 | PA325 | 3 | 180 | S | [8] |
| 43 | PA346 | 3 | 180 | S | [8] |
| 44 | PA60 | 3 | 180 | S | [8] |
| 45 | ^6B^-8 | 6B | 185 | NS | S. Africa ^6B^-8 |
| 46 | PA52 | 19A | 199 | S | [8] |
| 47 | PA136 | 19A | 199 | NS | [8] |
| 48 | PA223 | 15 | 199 | S | [8] |
| 49 | PA147 | 4 | 205 | S | [8] |
| 50 | PA382 | 1 | 227 | S | [8] |
| 51 | ^19F^-14 | 19F | 236 | NS | Taiwan ^19F^-14 |
| 52 | ^23F^-15 | 23F | 242 | NS | Taiwan ^23F^-15 |
| 53 | ^19A^-6 | 19A | 268 | NS | Hungary ^19A^-6 |
| 54 | ^6B^-12 | 6B | 270 | NS | Finland ^6B^-12 |
| 55 | ^5^-19 | 5 | 289 | S | Columbia ^5^-19 |
| 56 | ^6B^-20 | 6B | 315 | S | Poland ^6B^-20 |
| 57 | ^6A^-23 | 6A | 376 | NS | N. Carolina ^6A^-23 |
| 58 | ^35B^-24 | 35B | 377 | NS | Utah ^35B^-24 |
| 59 | PA73 | 6B | 377 | NS | [8] |
| 60 | PA74 | 35 | 377 | NS | [8] |
| 61 | PA99 | 14 | 377 | NS | [8] |
| 62 | ^6B^-17 | 6B | 384 | NS | Maryland ^6B^-17 |
| 63 | PA280 | 38 | 393 | S | [8] |
| 64 | PA200 | 22 | 433 | S | [8] |
| 65 | PA217 | 35 | 498 | S | [8] |
| 66 | PA183 | 16 | 659 | S | [8] |
| 67 | PA97 | 6A | 690 | S | [8] |
| 68 | PA169 | 19A | 690 | S | [8] |
| 69 | PA224 | NT | 690 | S | [8] |
| 70 | PA310 | 10 | 816 | S | [8] |
| 71 | PA185 | 7 | 1201 | S | [8] |
| 72 | PA179 | 20 | 1257 | S | [8] |

S: Susceptible, NS: Non-susceptible
