# Supplementary material for: Monitoring the Long-Term Molecular Epidemiology of the Pneumococcus and Detection of Potential ‘Vaccine Escape’ Strains
Source: PLoS One. 2011 Jan 10;6(1):e15950. doi: 10.1371/journal.pone.0015950 (PMC3018475; doi:10.1371/journal.pone.0015950)
Supplement: Table S2 — List of primers used for expansion of sequence coverage. (DOC) [file pone.0015950.s004.doc]

**Table S2: List of primers used for expansion of sequence coverage**

End primers:

| **Primer Name** | **Primer Sequence** | **Length (bp)** | **Tm** | **% GC** |
| --- | --- | --- | --- | --- |
| 16S_rRNAF | CCTAATACATGCAAGTAGAACGCTG | 25 | 61.32 | 44 |
| 16S_rRNAR | CCTTACGGTTACCTCACCGACTTCG | 25 | 67.73 | 56 |
| SP_1961F | TTGGCAGGACATGACGTTCAATACG | 25 | 68.98 | 48 |
| SP_1961R | TTCTTGTTCAGCAGCTTCTTCTGTT | 25 | 62.96 | 40 |
| SP_0117F | ATGAATAAGAAAAAAATGATTTTAA | 25 | 53.06 | 12 |
| SP_0117R | AACCCATTCACCATTGGCATTGACT | 25 | 68.4 | 44 |
| SP_0368F | ATGAATAAAGGATTATTTGAAAAAC | 25 | 54.38 | 20 |
| SP_0368R | GTCTTTCTTCGTTTTTACTACAAAG | 25 | 55.2 | 32 |
| SP_0667F | ATGAATAAACGTCTATTTTCAAAAA | 25 | 55.65 | 20 |
| SP_0667R | ATCATCTAAATGATCAATGGCATAT | 25 | 57.92 | 28 |
| SP_0834F | GTGTCAGCACAAATTACGATTAACC | 25 | 60.98 | 40 |
| SP_0834R | CAAGACATCATCGTCACTCACCTTG | 25 | 65.22 | 48 |
| SP_1204F | TTGCAGGTCTTTGATTTGTCGGTGG | 25 | 69.99 | 48 |
| SP_1204R | TTCATTTTTAAATTGACTATGCGCC | 25 | 62.41 | 32 |
| SP_1466F | ATGAACACTAGTCTTAAACTCAGCA | 25 | 56.61 | 36 |
| SP_1466R | CATGTAATAAACAATAGCGATGTAT | 25 | 54.63 | 28 |
| SP_1833F | ATGAAATATTTTGTTCCTAATGAGG | 25 | 56.97 | 28 |
| SP_1833R | TTTATCTTTACCATAATACTTCAAC | 25 | 51.26 | 24 |
| SP_1992F | GTGACGATTCTAGGAAAAGATACAG | 25 | 57.67 | 40 |
| SP_1992R | TTTTCTACGTCTAGTCTTTCTTCCT | 25 | 56.45 | 36 |
| SP_2145F | ATGAAACCACTACTTGAAACCATCG | 25 | 62.54 | 40 |
| SP_2145R | GTGACTTGGTAACCAGCTGAGGGTG | 25 | 67.19 | 56 |

**Gene-specific walking primers**

**SP_1466 : Hemolysin**

| **S. No.** | **Primer Sequence** | **Length (bp)** | **Tm** | **% GC** |
| --- | --- | --- | --- | --- |
| 1 | ATGAACACTAGTCTTAAACTCAGC | 24 | 53.97 | 38 |
| 2 | CATGTAATAAACAATAGCGATGTATTG | 27 | 58.22 | 30 |

**SP_1204 : Hemolysin A - putative**

| **S. No.** | **Primer Sequence** | **Length (bp)** | **Tm** | **% GC** |
| --- | --- | --- | --- | --- |
| 1 | GCCGTGGTGGTTTGAAACTGGAAAAG | 26 | 70.93 | 50 |
| 2 | TTCATTTTTAAATTGACTATGCGCC | 25 | 62.41 | 32 |

**SP_0834 : Hemolysin-related protein**

| **S. No.** | **Primer Sequence** | **Length (bp)** | **Tm** | **% GC** |
| --- | --- | --- | --- | --- |
| 1 | GTGTCAGCACAAATTACGATTAAC | 24 | 57.84 | 38 |
| 2 | GATTACAAGACATCATCGTCAC | 22 | 54.63 | 41 |

**SP_1961 : DNA-directed RNA polymerase β subunit**

| **S. No.** | **Primer Sequence** | **Length (bp)** | **Tm** | **% GC** |
| --- | --- | --- | --- | --- |
| 1 | GATTAGAAAAGCCTGATTGACAAG | 24 | 58.53 | 38 |
| 2 | TTCTTGTTCAGCAGCTTCTTCTG | 23 | 61.19 | 43 |
| 3 | CCGTCGTAGTTTTTCAAGA | 19 | 53.96 | 42 |
| 4 | GTTTCAGGGTCTACCAATG | 19 | 52.83 | 47 |
| 5 | TCGTTTCTCAGTTGGTCC | 18 | 54.34 | 50 |
| 6 | GAAATCGTCGTATCCGTG | 18 | 54.84 | 50 |
| 7 | GCGATGTAAACACGAACC | 18 | 54.92 | 50 |
| 8 | GGTCGGAGATAAAATGGC | 18 | 55.47 | 50 |
| 9 | GGCTTTTTCAAGGTCATC | 18 | 53.02 | 44 |
| 10 | CTTTTGTTGCTTTCTCAGC | 19 | 53.8 | 42 |
| 11 | CCGTTATCATGAGCGAAC | 18 | 55.02 | 50 |
| 12 | TGGGACTTTCTCGTATGG | 18 | 54.34 | 50 |
| 13 | AAGATGCCCTCAAAGACC | 18 | 55.06 | 50 |
| 14 | TTGGAGTCATCGTTTTCC | 18 | 54.29 | 44 |
| 15 | AAATCTTGGTAGAAGCTGG | 19 | 52.05 | 42 |
| 16 | ATCATCTACACGGCCAAG | 18 | 54.37 | 50 |
| 17 | AGGCCCAAGCTTTGTATC | 18 | 55.29 | 50 |
| 18 | CAACGTTTCGGTGAGATG | 18 | 55.95 | 50 |
| 19 | TGCTTCTTTAACAGTTGACC | 20 | 53.08 | 40 |
| 20 | ATTCCTTCATCCAAGTCG | 18 | 53.29 | 44 |
| 21 | GCTTCTTCAGCTTCAAAGG | 19 | 55.4 | 47 |
| 22 | AAGTAAACACCTGGTGAGC | 19 | 52.64 | 47 |
| 23 | GGATATGAAATCAAGGAACC | 20 | 53.53 | 40 |

**SP_1833 : Cell wall surface anchor family protein**

| **S. No.** | **Primer Sequence** | **Length (bp)** | **Tm** | **% GC** |
| --- | --- | --- | --- | --- |
| 1 | ATGAAATATTTTGTTCCTAATGAGGTATTCAG | 32 | 62.87 | 28 |
| 2 | TTTATCTTTACCATAATACTTCAACCC | 27 | 57.45 | 30 |
| 3 | CGATGGCTACAAAAGAGTC | 19 | 53.38 | 47 |
| 4 | CTATTCAAATTGCAGGTTCG | 20 | 55.99 | 40 |
| 5 | TTATACGTGATAGCGATGG | 19 | 52.18 | 42 |
| 6 | TCAGTGAATCCTGTAAAACG | 20 | 53.79 | 40 |
| 7 | CTTCAACCCTTTTAGTGTCA | 20 | 52.93 | 40 |
| 8 | CAATCGTACCACCAGAATAAC | 21 | 54.70 | 43 |
| 9 | TAGCAAATGCACCTGAAG | 18 | 53.18 | 44 |
| 10 | AAACCAATTCCTGTCACTG | 19 | 53.33 | 42 |
| 11 | AGGTGCATTTGCTATTGG | 18 | 54.59 | 44 |
| 12 | ATGGTGCGCAAGTAGAAT | 18 | 54.11 | 44 |
| 13 | GCCATCGGTGAAGAACTA | 18 | 54.51 | 50 |

**SP_1992 : Cell wall surface anchor family protein**

| **S. No.** | **Primer Sequence** | **Length (bp)** | **Tm** | **% GC** |
| --- | --- | --- | --- | --- |
| 1 | GTGACGATTCTAGGAAAAGATACAG | 25 | 57.67 | 40 |
| 2 | TTTTCTACGTCTAGTCTTTCTTCC | 24 | 55.46 | 38 |

**SP_0368 : Cell wall surface anchor family protein**

| **S. No.** | **Primer Sequence** | **Length (bp)** | **Tm** | **% GC** |
| --- | --- | --- | --- | --- |
| 1 | GGATTTAGAAGTTTATGAATAAAGGATTATTTG | 33 | 61.21 | 24 |
| 2 | GTCTTTCTTCGTTTTTACTACAAAG | 25 | 55.2 | 32 |
| 3 | CCAATGGAAATGCAACTG | 18 | 55.86 | 44 |
| 4 | TTGGGGTAATGGTCAATC | 18 | 53.91 | 44 |
| 5 | CCAATGGAAATGCAACTG | 18 | 55.86 | 44 |
| 6 | CATAGATAAAGTCGAGACCG | 20 | 52.58 | 45 |
| 7 | CCTGTTAGGGTAATTTCTTCG | 21 | 55.67 | 43 |
| 8 | GGGTTACATGTATGGATTTG | 20 | 52.78 | 40 |
| 9 | TTCCGAGCAAGGTAAGAC | 18 | 53.77 | 50 |
| 10 | AAAGTCTTTCATGCTGGAG | 19 | 52.88 | 42 |
| 11 | CTGTTGCTGGCAAGATTT | 18 | 54.79 | 44 |
| 12 | GTATTGTTTGACATGGTTGC | 20 | 53.89 | 40 |
| 13 | GGAGTCAAGTAAGCTGAACC | 20 | 54.04 | 50 |
| 14 | AACAGAAGCAACACCTAATG | 20 | 52.95 | 40 |
| 15 | CATTAGGTGTTGCTTCTGTT | 20 | 52.95 | 40 |
| 16 | CTGGTGATCAAGCAACTTAC | 20 | 53.32 | 45 |
| 17 | GAAGAAGTGAATACAATGGG | 20 | 52.15 | 40 |

**SP_2145 : Antigen, cell wall surface anchor family protein**

| **S. No.** | **Primer Sequence** | **Length (bp)** | **Tm** | **% GC** |
| --- | --- | --- | --- | --- |
| 1 | GAAACCACTACTTGAAACCATC | 22 | 55.4 | 41 |
| 2 | GTTTCATAAAAGAAGAACCTTTGC | 24 | 57.7 | 33 |
| 3 | AAAGTAGCTTGAATGGCAG | 19 | 53.19 | 42 |
| 4 | CAGGATGGATCTTGGTTTT | 19 | 54.93 | 42 |
| 5 | GTGGAAAAGAAACCTTTAGC | 20 | 53.4 | 40 |
| 6 | TCAGCGTCAAATAGCTGG | 18 | 55.93 | 50 |
| 7 | AAGAGTCTGTAGAGGCAGTG | 20 | 52.05 | 50 |
| 8 | AGATCCATCCTGGTCACT | 18 | 52.26 | 50 |

**SP_0667 : Pneumococcal surface protein - putative**

| **S. No.** | **Primer Sequence** | **Length (bp)** | **Tm** | **% GC** |
| --- | --- | --- | --- | --- |
| 1 | GGAGAAAGAAATGAATAAACGTC | 23 | 56.21 | 35 |
| 2 | GTTAATCATCTAAATGATCAATGGC | 25 | 58.07 | 32 |
| 3 | AGCTTGATAGGTGGCATC | 18 | 53.5 | 50 |
| 4 | CCTTTCCTAAAGCAGCAG | 18 | 53.75 | 50 |
| 5 | TCAACCCATTCATTGACAGC | 20 | 59.5 | 45 |
| 6 | GACGTTATTATGTTGGCG | 18 | 52.43 | 44 |

**SP_0117 : Pneumococcal surface protein A**

| **S. No.** | **Primer Sequence** | **Length (bp)** | **Tm** | **% GC** |
| --- | --- | --- | --- | --- |
| 1 | CTATCAGAAAAGAGGTAAATTTAGATG | 27 | 55.66 | 30 |
| 2 | CCTTATCTTTGTTTCAAAATTAAAATGTC | 29 | 55.98 | 24 |
| 3 | TATCTTAGGGGCTGGTTT | 18 | 52.25 | 44 |
| 4 | GCACTAGCGAAGAAAGAAGTAG | 22 | 55.54 | 45 |
| 5 | CCTAACTCATTAAGAGCTGC | 20 | 52.08 | 45 |
| 6 | GTTGTGTTGACTGCAAGG | 18 | 53.12 | 50 |
| 7 | CCACATACCGTTTTCTTG | 18 | 52.22 | 44 |
| 8 | AAACGCTAAACAAGCTGAG | 19 | 53.46 | 42 |
| 9 | ATGATACAGAAGCTATAGAAG | 21 | 45.8 | 33 |
| 10 | ATGAAGAAGAAACTCCAGCG | 20 | 57.14 | 45 |
| 11 | ATCTGTTCGAGCAATGGTAG | 20 | 55.41 | 45 |
| 12 | AGATAAAGAAGCTGCTGAAG | 20 | 51.77 | 40 |
| 13 | ATTAGATGATGCATCAGCTC | 20 | 52.78 | 40 |
| 14 | GAAGAAGAAACTCCAGCG | 18 | 53.44 | 50 |
| 15 | AAGAAGCAGAAGTAGCTAAG | 20 | 48.7 | 40 |
| 16 | ATGAAGAAGAGACTCCAGC | 19 | 51.72 | 47 |
| 17 | TTGCTCTGGTTGAGGAGC | 18 | 57.51 | 56 |
| 18 | TCGTCTACTTTCTTAGCAGC | 20 | 52.7 | 45 |
| 19 | ATTCTCAGGCTCTTCAGC | 18 | 52.82 | 50 |
| 20 | TATCTTAGGGGCTGGTTT | 18 | 52.25 | 44 |
| 21 | GTTGTGTTGACTGCAAGG | 18 | 53.12 | 50 |
| 22 | CCTAACTCATTAAGAGCTGC | 20 | 52.08 | 45 |
| 23 | GCATCATAGTCTTTCTCAGC | 20 | 52.57 | 45 |
| 24 | CTTCAAAACAAAGTCGCTG | 19 | 54.63 | 42 |
| 25 | TAGAGGCAGAAGAAGCTGAG | 20 | 55.16 | 50 |
| 26 | TATCGCGACTAAACAAGC | 18 | 52.48 | 44 |
| 27 | GGATGCTGAAAAATATGCTC | 20 | 54.96 | 40 |
| 28 | GGAGCTTCTTCTGCTCTTAC | 20 | 53.6 | 50 |
| 29 | TGACTTTAGCCCAACCTG | 18 | 54.63 | 50 |
| 30 | AAGATTGATGAGTTAGACGC | 20 | 52.03 | 40 |
| 31 | TCGAGCAATGGTAGTTCC | 18 | 54.51 | 50 |
| 32 | TAAAGAAGGTCTCCGTGC | 18 | 53.77 | 50 |
| 33 | GGCTGGTTTTGTTACGTCT | 19 | 55.3 | 47 |
| 34 | AAGTCTAGCCAGCGTCGC | 18 | 59.3 | 61 |
| 35 | TTCTAGATTCTCGTAATAGC | 20 | 46.67 | 35 |
| 36 | ATGCAACTCTAAAGGTAGC | 19 | 49.66 | 42 |
| 37 | AAGAGTCTGTAGAGGCAGTG | 20 | 52.05 | 50 |
| 38 | GAATTGGATGTTAAGCAAGC | 20 | 55.05 | 40 |
