# Supplementary material for: Monitoring the Long-Term Molecular Epidemiology of the Pneumococcus and Detection of Potential ‘Vaccine Escape’ Strains
Source: PLoS One. 2011 Jan 10;6(1):e15950. doi: 10.1371/journal.pone.0015950 (PMC3018475; doi:10.1371/journal.pone.0015950)
Supplement: Table S3 — Gene loci and strains with <95% sequence coverage. (DOC) [file pone.0015950.s005.doc]

**Table S**3: Gene loci and strains with < 95% sequence coverage

| **S. No** | **Locus** | **Gene/sequence** | **Length (BP)** | **Strain** | **Serotype/Serotype** | **ST-complex** | **Pen susceptibility** |
| --- | --- | --- | --- | --- | --- | --- | --- |
| 1 | SP_0117 | Pneumococcal surface protein A | 2232 | PA195 | 23B | 81 | NS |
| 2 |  |  |  | PA189 | 19F | 81 | NS |
| 3 |  |  |  | ^23F^-16 | 23F | 173 | NS |
| 4 |  |  |  | PA280 | 38 | 393 | S |
| 5 | SP_0667 | Pneumococcal surface protein-putative | 996 | ^23F^-1 | 23F | 81 | NS |
| 6 |  |  |  | PA189 | 19F | 81 | NS |
| 7 |  |  |  | ^6B^-8 | 6B | 185 | NS |
| 8 | SP_368 | Cell wall surface anchor family protein 1 | 5301 | ^14^-9 | 14 | 9 | S |
| 9 |  |  |  | ^14^-10 | 14 | 20 | NS |
| 10 |  |  |  | ^19A^-11 | 19A | 175 | NS |
| 11 | SP_1833 | Cell wall surface anchor family protein 2 | 2124 | PA230 | 3 | 180 | S |
