# Supplementary material for: Monitoring the Long-Term Molecular Epidemiology of the Pneumococcus and Detection of Potential ‘Vaccine Escape’ Strains
Source: PLoS One. 2011 Jan 10;6(1):e15950. doi: 10.1371/journal.pone.0015950 (PMC3018475; doi:10.1371/journal.pone.0015950)
Supplement: Table S4 — Validation of resequencing array based on ability to detect SNPs. (DOC) [file pone.0015950.s006.doc]

**Table S4: Validation of resequencing array based on ability to detect SNPs**

| **Gene Classification** | **Name/**  **Locus** | **Gene/ Sequence** | **Sequenced fragment length  (bp)** | **TIGR4** | | | | | **R6** | | | | | **670** | | | | |
| --- | --- | --- | --- | --- | --- | --- | --- | --- | --- | --- | --- | --- | --- | --- | --- | --- | --- | --- |
|  |  |  |  | **Total filtered calls** | **Predicted SNPs** | **TP  SNP**  **calls** | **FP SNP calls** | **FN SNP calls** | **Total filtered calls** | **Predicted SNPs** | **TP SNP calls** | **FP SNP calls** | **FN SNP calls** | **Total filtered calls** | **Predicted SNPs** | **TP SNP calls** | **FP SNP calls** | **FN SNP calls** |
| Conserved | 16S_rRNA | 16S rRNA | 1389 | 1379 | 0 | 0 | 0 | 0 | 1380 | 3 | 3 | 0 | 0 | 1377 | 3 | 3 | 0 | 0 |
|  | SP_0834 | Hemoylsin-related protein | 486 | 478 | 0 | 0 | 0 | 0 | 478 | 6 | 6 | 0 | 0 | 455 | 8 | 7 | 0 | 1 |
|  | SP_1204 | Hemolysin A-putative | 570 | 554 | 0 | 0 | 0 | 0 | 543 | 4 | 4 | 0 | 0 | 565 | 1 | 1 | 0 | 0 |
|  | SP-1466 | Hemoylsin | 621 | 615 | 0 | 0 | 0 | 0 | 612 | 1 | 1 | 0 | 0 | 598 | 3 | 1 | 0 | 2 |
|  | SP_1961 | DNA-directed RNA pol-β subunit | 3585 | 3491 | 0 | 0 | 0 | 0 | 3498 | 21 | 17 | 0 | 4 | 3394 | 24 | 18 | 0 | 6 |
| Variable | SP_2145 | Antigen, cell wall surface anchor | 2058 | 2017 | 0 | 0 | 0 | 0 | 1867 | 31 | 11 | 1 | 20 | 1836 | 39 | 13 | 0 | 26 |
|  | SP_0368 | Cell wall surface anchor family protein 1 | 5277 | 5094 | 0 | 0 | 0 | 0 | 4702 | 94 | 53 | 0 | 41 | 4348 | 138 | 37 | 2 | 101 |
|  | SP_1833 | Cell wall surface anchor family protein 2 | 2100 | 1930 | 0 | 0 | 0 | 0 | 1933 | 6 | 4 | 0 | 2 | 1878 | 5 | 3 | 0 | 2 |
|  | SP_1992 | Cell wall surface anchor family protein 3 | 639 | 611 | 0 | 0 | 0 | 0 | 620 | 1 | 1 | 0 | 0 | 574 | 8 | 6 | 0 | 2 |
|  | SP_0667 | Pneumococcal surface protein - putative | 972 | 956 | 0 | 0 | 0 | 0 | 941 | 4 | 3 | 0 | 1 | 335 | 11 | 2 | 0 | 9 |
|  | SP_0117 | Pneumococcal surface protein A | 2208 | 2133 | 0 | 0 | 0 | 0 | 760 | 29 | 4 | 5 | 25 | 792 | 9 | 0 | 5 | 9 |
|  |  | **Total** | **19905** | **19258** | **0** | **0** | **0** | **0** | **17334** | **200** | **107** | **6** | **93** | **16152** | **249** | **91** | **7** | **158** |

Known genome sequences of TIGR4, R6 and 670 were used to predict the expected SNPs and determine true and false positives. A higher number of false negatives, especially in variable genomic regions can be attributed to the sequence diversity along with the stringent filter parameters used in our approach [44].

TP: True positive; FN: False negative, these are the expected SNP calls which were not called at expected SNP locations; FP: False positive calls are SNP calls that occurred at unexpected locations.
